# Increasing grades of frontal deformities in knee osteoarthritis are not associated with ligamentous ankle instabilities

**DOI:** 10.1007/s00167-022-07021-3

**Published:** 2022-06-06

**Authors:** F. Graef, M. Rühling, C. Gwinner, H. Hommel, S. Tsitsilonis, C. Perka

**Affiliations:** 1 Center for Musculoskeletal Surgery, Charité – Universitätsmedizin Berlin, corporate member of Freie Universität Berlin and Humboldt-Universität zu Berlin, Charitéplatz 1, 10117 Berlin, Germany; 2 Department of Orthopaedics, Märkisch-Oderland Hospital, Brandenburg Medical School Theodor Fontane, Wriezen, Germany

**Keywords:** Total knee arthroplasty, Knee osteoarthritis, Ankle stability, Kinematic chain, Joint interaction, Frontal plane alignment, Coronal alignment

## Abstract

**Purpose:**

Varus or valgus deformities in knee osteoarthritis may have a crucial impact on ankle subtalar range of motion (ROM) and ligamentous stability. The purpose of this study was to assess whether the grade of ankle eversion and inversion rotation stability was influenced by frontal deformities of the knee joint.

**Methods:**

Patients who were planned to undergo total knee arthroplasty (TKA) were prospectively included in this study. Patients were examined radiologically (mechanical tibiofemoral angle (mTFA), hindfoot alignment view angle (HAVA), anterior distal tibia angle (ADTA)) and clinically (ROM of the knee and ankle joint, foot function index, knee osteoarthritis outcome score). Ankle stability was assessed using an ankle arthrometer (AA) to test inversion/eversion (ie) rotation and anterior/posterior (ap) displacement stability of the ankle joint. Correlations were calculated using Pearson’s coefficient, and differences between two independent groups of nonparametric data were calculated using a two-sided Wilcoxon signed rank test.

**Results:**

Eighty-two (varus *n = *52, valgus *n = *30) patients were included. The preoperative mTFA significantly correlated with the HAVA (Pearson’s correlation* = *− 0.72, *p < *0.001). Laxity testing of the ankle demonstrated that in both varus and valgus knee osteoarthritis, higher grades of mTFA did not correlate with the inversion or eversion capacity of the ankle joint. The ADTA significantly correlated with the posterior displacement of the ankle joint (cor = 0.24, *p = *0.049).

**Conclusions:**

This study could not confirm that higher degrees of frontal knee deformities in osteoarthritis were associated with increasing grades of ligamentous ankle instabilities or a reduced ROM of the subtalar joint.

**Level of evidence:**

II.

## Introduction

Frontal varus or valgus deformities in end-stage osteoarthritis of the knee can be corrected by total knee arthroplasty (TKA) to a neutral mechanical alignment.

[37]. However, when a joint is mechanically altered, this can also have an impact on neighboring joints [21, 36]. Recent studies claimed that TKA can lead to increased ankle symptoms [10, 34]. Radiologic analyses have demonstrated that the subtalar joint partially compensates for varus malalignments at the knee by valgization and valgus deformities at the knee by varization [2, 28]. It was, therefore, suggested that if the subtalar joint became stiff, the compensation mechanism fails, consequently leading to increased ankle pain [24, 33].

These studies were conducted retrospectively by merely analysing changes in the hindfoot on X-rays. These reports did not examine the hindfoot range of motion or instabilities at the ankle joint. Therefore, it remains speculative whether a stiff subtalar joint is responsible for the onset of ankle symptoms after TKA. Frontal malalignments at the knee joint can vastly impact the ligamentous balance of the knee [3]. It remains unknown to what extent ligamentous balancing at the ankle joint is influenced by valgus or varus knee osteoarthritis. The purpose of this study was, therefore, to measure the ankle stability and range of motion of the subtalar joint in patients with osteoarthritis of the knee and an additional frontal deformity before undergoing TKA.

The main hypothesis of this study was, therefore, that higher degrees of the preoperative mechanical tibiofemoral angle (mTFA) (varus malalignment) were associated with higher degrees of eversion and lower degrees of inversion at the ankle joint. Conversely, lower degrees of mTFA (valgus malalignment) were associated with higher degrees of inversion and lower degrees of eversion at the ankle joint. In addition to the main hypothesis, the aim was to report whether patients with high-grade frontal knee deformities had a reduced range of motion (ROM) of the subtalar joint. Furthermore, correlation analyses were calculated to unveil associations between the mTFA and the anterior distal tibia angle (ADTA), between the knee slope and ADTA and between the ap (anterior–posterior) displacement and the knee slope.

## Methods

This study was approved by the local ethics committee (approval number: AS 116(bB)/2019). Written informed consent was obtained from all patients. The study protocol was registered at the German Clinical Trials Register (DRKS-ID: DRKS00017400).

From September 2020 until September 2021, patients with osteoarthritis of the knee who were scheduled to undergo TKA were asked to participate in this study. The inclusion criteria were planned TKA for primary osteoarthritis of the knee, age > 18 years, willingness to participate, and all sexes. The exclusion criteria were rheumatoid arthritis, previous hindfoot operations or joint fusions of the foot and ankle, posttraumatic pathologies/osteoarthritis of the foot and ankle joint, neurologic disorders or polyneuropathy affecting gait and postural control (e.g. Parkinson disease), progressed diabetes, and Charcot’s foot. For subanalyses, patients were subdivided into four categories according to their preoperative mTFA: 0–5°, 5–10°, 10–15° and > 15°, as previously reported [7].

The study was conducted at a German university hospital with a board-certified joint replacement center. The PROSPERO checklist for reporting observational studies was followed for this clinical prospective study [5].

### Clinical examination

One day preoperatively, patients were clinically examined by measuring the ROM of the knee and ankle joint. The ROM of the ankle joint was measured with the knee in 90° flexion. For statistical reasons, a motion deficit of, e.g. a 5° extension deficit of the knee (extension/flexion 0–5–90°) was documented as extension = − 5°. Subjective patient satisfaction was assessed using patient-related outcome measures (PROMs). At the knee joint, the knee osteoarthritis outcome score (KOOS) was used [19]. The function of the ankle joint was reported using the American Orthopedic Foot and Ankle Score for the hindfoot (AOFAS) [23]. Patient satisfaction concerning the foot and ankle was assessed with the Foot Function Index (FFI) [26].

### Radiologic analysis

**Fig. 1:**
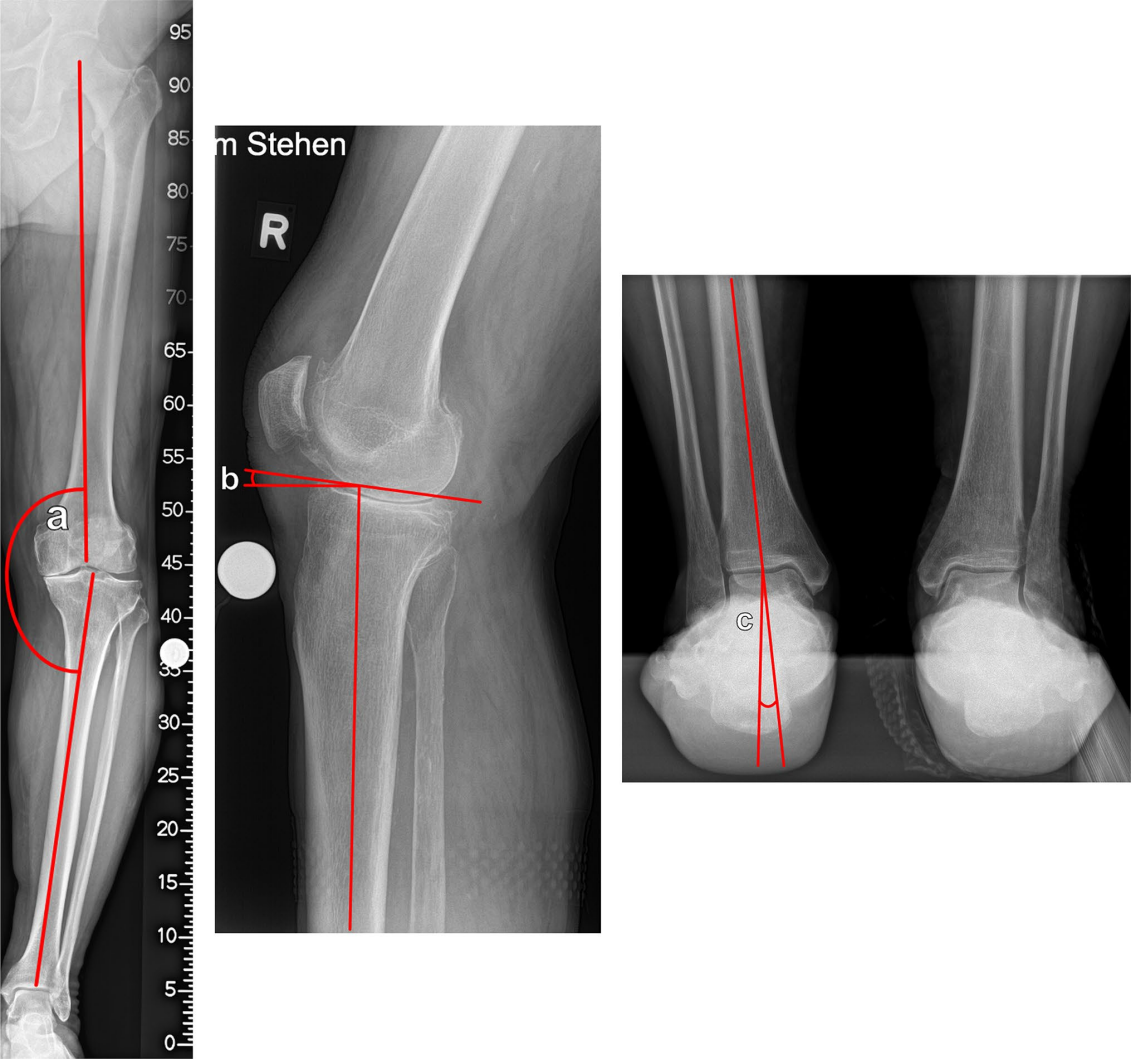
**a** The mTFA (**a**) was defined as the angle between the mechanical femoral and mechanical tibial axes. The mLDFA (**b**) was the angle between a tangent to the subchondral distal femoral condyles and the mechanical femoral axis. The MPTA (**c**) was the angle between the tangent to the proximal tibial joint surface and the mechanical tibial axis. **b** The slope (**d**) was measured as suggested by Dejour et al. [4, 11]. **c** The HAVA (**e**) was the intersection between the mechanical tibial axis and a line connecting the most distal point of the calcaneus and the centre of the ankle joint in a standing hindfoot radiograph

**Fig. 2:**
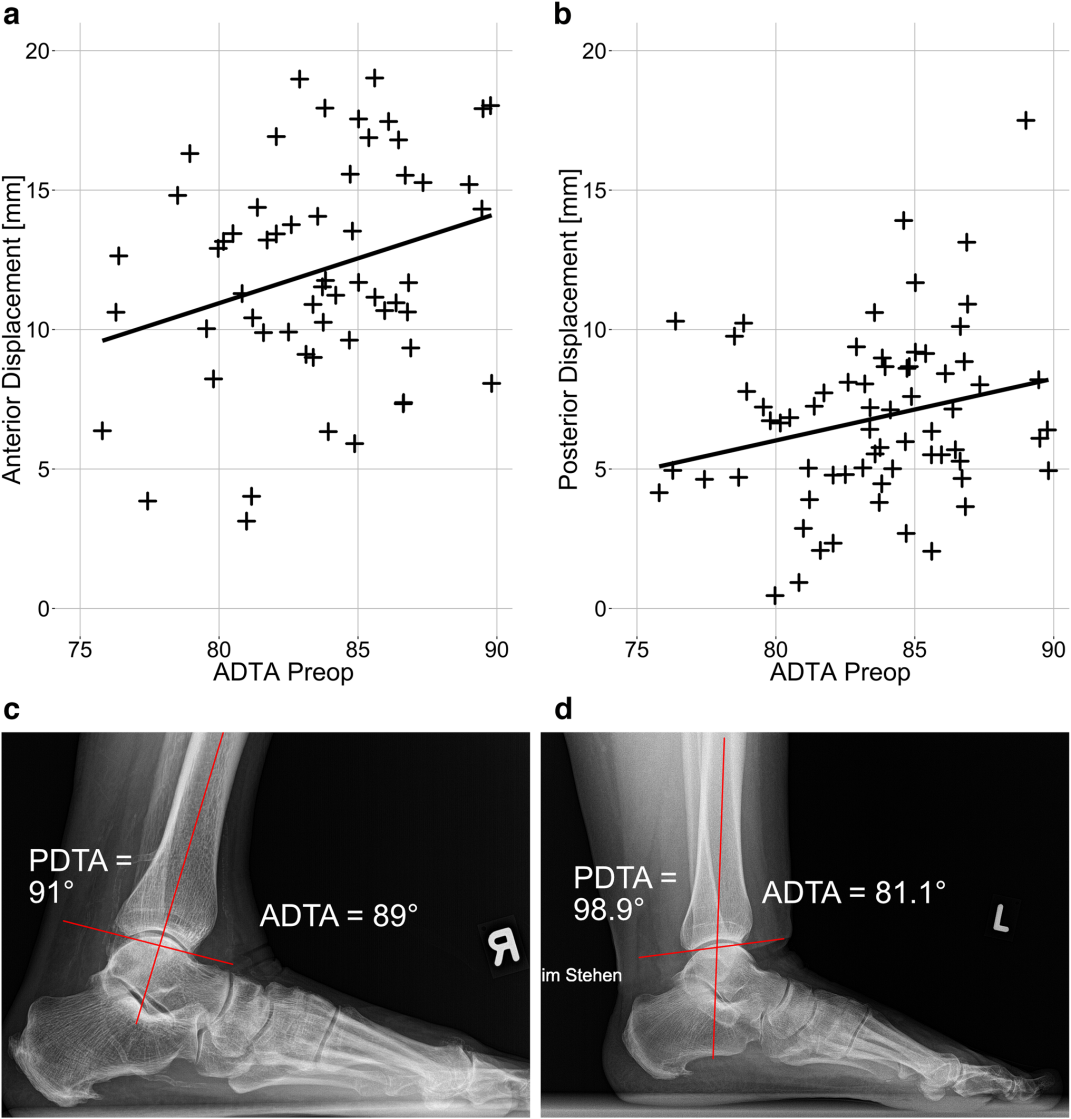
**a** The ADTA positively correlated with anterior displacement of the talus, although this correlation was not significant (Pearson’s cor* = *0.147, *p = *n.s.). **b** The posterior displacement significantly correlated with the ADTA (Pearson’s cor = 0.24, *p = *0.049). **c** Lateral X-ray of a standing ankle and foot with the ADTA being measured as nearly horizontal (~ 90°). The posterior displacement of the talus of this patient was up to 17.5 mm. **d** X-ray of a patient with an ADTA = 81.1° and a subsequently increased PDTA of 98.9°. This patient had a posterior displacement of 5.2 mm. *ADTA* anterior distal tibia angle, *PDTA* posterior distal tibia angle. Significance level *p < *0.05

Preoperative X-rays were acquired with the patient standing upright and full weight bearing and included the following: an anterior–posterior radiograph of the entire leg with the knee in a neutral position and the patella facing anteriorly as well as a lateral view of the knee only. If patients had a preoperative varus/valgus ≥ 5°, additional X-rays of the foot and ankle were taken as follows: a lateral view of the entire foot, including the ankle joint, a mortise view of the ankle joint and a hindfoot view [31]. The following mechanical angles were measured using X-rays: the mTFA is the intersection between the mechanical femoral axis and the mechanical tibial axis measured in X-rays of the entire standing leg [1]. The mechanical lateral distal femur angle (mLDFA) was the angle between the tangent to the subchondral distal femoral condyles and the mechanical femoral axis. The MPTA was the angle between the tangent to the proximal tibial joint surface and the mechanical tibial axis [14]. The slope was measured as suggested by Dejour et al. [4, 11]. The hindfoot alignment view angle (HAVA) is the intersection between the mechanical tibial axis and a line that connects the most distal part of the calcaneus and the center of the ankle joint (Fig. 1) [2, 31]. In all measurements, positive values correspond to varus alignment, and negative values correspond to valgus alignment. The ADTA is the “slope” of the ankle joint and is constructed by the mechanical tibial axis and a subchondral tangent to the tibia plafond connecting the most anterior and most posterior cortex on lateral radiographs of the ankle joint (Fig. 2) [18]. All radiologic measurements were performed by two independent experienced observers with more than five years of musculoskeletal imaging experience at two time points with a minimum of 14 days between measurements to calculate inter- and intraobserver reliability.

### Ankle arthrometer

**Fig. 3:**
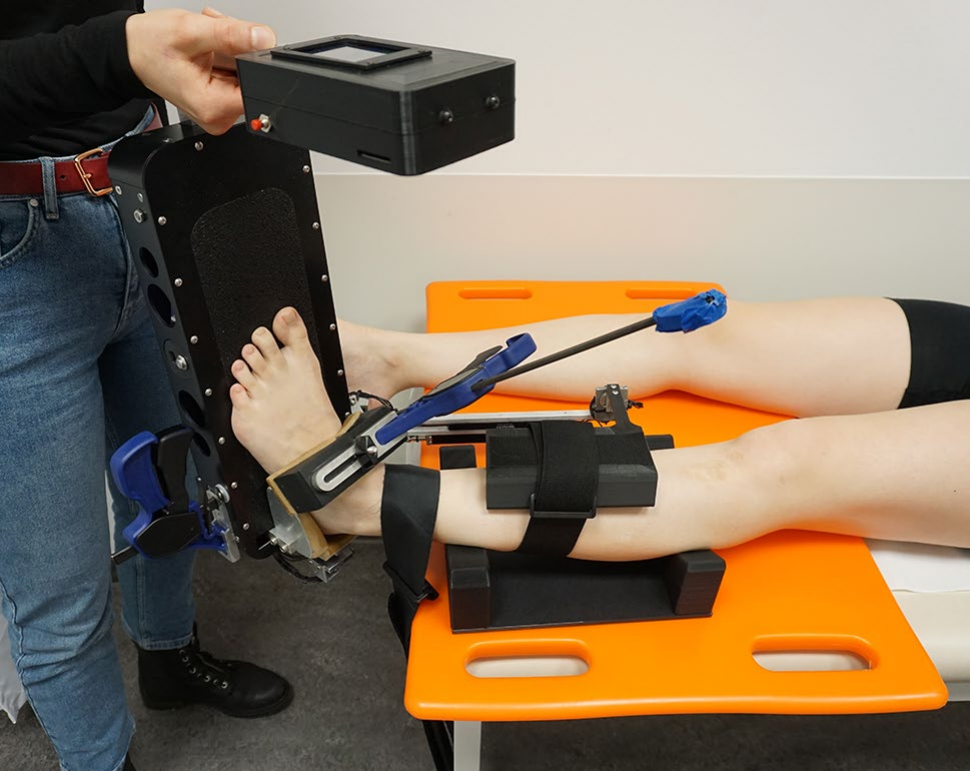
Photo of the ankle arthrometer (Hollis Ankle Arthrometer™, Blue Bay Research, Inc., Florida, USA) and the experimental setup (**a**). The patient is lying on a stretcher, and the leg is positioned in the calf support on a hard pad and is fixed to the table proximal to the ankle joint. In this photo, an inversion test is being performed

Ligamentous ankle stability was assessed using an AA (Hollis Ankle Arthrometer™, Blue Bay Research, Inc., Florida, USA). All AA measurements were performed at one time point by a single investigator who was trained for this device. The arthrometer has been reported to be the most widely used for measuring ankle laxity and has high intra- and intertester reliability [13, 16]. For the measurement, patients were in a prone position on a stretcher. The examined leg was placed in the calf support and additionally positioned on a hard support pad to minimise measuring inaccuracies possibly caused by the soft padding of the stretcher. The distal tibia was strapped to the table, and measurement of the maximum inversion–eversion (ie) rotation as well as maximum anterior–posterior (ap) displacement was performed according to the manufacturer’s instructions with a neutrally aligned ankle joint in 0° dorsi/plantar flexion, 0 mm ap displacement and 0° ie rotation (Fig. 3). For the ap-measurement, a maximum force of 125 °N was applied, and for the ie testing, 4 Nm torque was applied. Measurements were stopped before reaching the maximum force if patients signalled that they experienced pain.

### Statistics

Statistics were performed using “R” and the software RStudio^©^ (RStudio, Inc., Boston, USA). Sample size calculation was conducted using the “pwr” package. For a medium effect size of *r = *0.3 and a power of 80%, *n = *82 patients were required to answer the main hypothesis. Data were analysed concerning normal/nonnormal distribution using histograms, QQ plots, mean/median and skewness. Correlations were displayed with scatter plots and calculated using Pearson’s (continuous data) correlation coefficient. Differences between two independent groups with nonnormal distribution were calculated using two-sided Wilcoxon signed rank tests. Independent categorial variables were tested using Fisher’s exact test. The inter- and intrarater reliability was calculated by the intraclass correlation coefficient (ICC) for continuous data using the “irr” package applying a two-way mixed effects model, absolute agreement and single unit type [22]. The significance level was *p < *0.05. Bonferroni correction was applied for multiple comparisons.

## Results

**Fig. 4:**
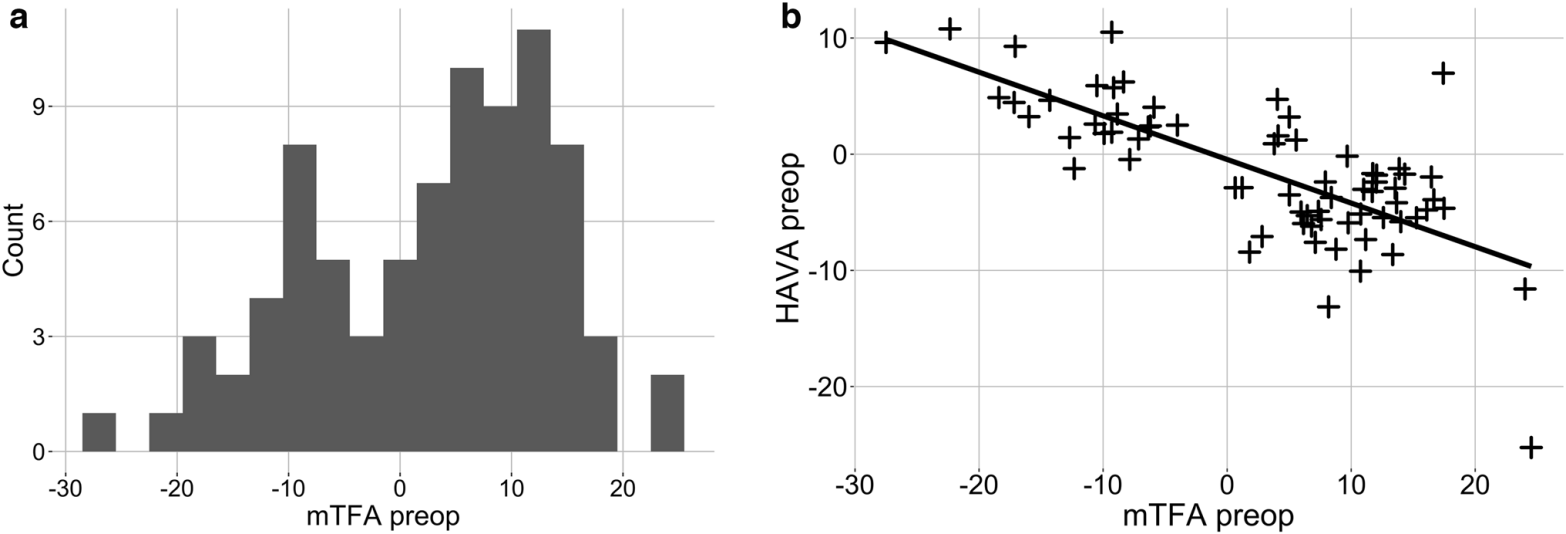
A total of 82 patients (varus *n* = 52, valgus *n* = 30) were included in this study, and the histogram in (**a**) depicts the frequency of preoperative mTFA malalignments. The preoperative mTFA was significantly and negatively correlated with the preoperative HAVA (Pearson’s cor =  − 0.72, *p < *0.001). Therefore, higher varus deformities at the knee joint were associated with higher grades of valgus at the hindfoot, and vice versa. *mTFA* mechanical tibiofemoral angle, *HAVA* hindfoot alignment view angle

Eighty-seven patients were asked to participate in this study. One patient had to be excluded due to previous hind- and forefoot fusion operations. Four patients were excluded since the surgeon decided to implant a unicondylar knee replacement during the operation. Subsequently, a total of 82 patients (varus *n = *52, valgus *n = *30) were included in this study (Fig. 4). Patient baseline characteristics and results from the PROMs are displayed in Table 1.

**Table 1:** Baseline characteristics and preoperative results of the clinical examination as well as patient related outcome measures

	Valgus	Varus	*p*
*n*	30	52	
BMI [median (IQR)]	25.8 [23.2, 30.3]	30.3 [21.1, 35.4]	0.004
Sex (%)			
M	5 (16.7)	26 (50.0)	0.006
W	25 (83.3)	26 (50.0)	
Side (%)			
Left	9 (30.0)	23 (44.2)	0.300
Right	21 (70.0)	29 (55.8)	
Groups (mTFA in °) (%)			
0–5°	6 (20.0)	9 (17.3)	0.675
5–10°	13 (43.3)	19 (36.5)	
10–15°	5 (16.7)	15 (28.8)	
> 15°	6 (20.0)	9 (17.3)	
ROM ankle dorsiflexion [median (IQR)]	5.0 [0.8, 10.0]	5.0 [0.0, 10.0]	0.992
ROM ankle plantarflexion [median (IQR)]	35.0 [30.0, 40.0]	35.0 [30.0, 40.0]	0.707
ROM knee extension [median (IQR)]	− 10.0 [− 13.8, 0.0]	− 5.0 [-10.0, 0.0]	0.053
ROM knee flexion [median (IQR)]	110.0 [90.0, 118.8]	115.0 [103.8, 120.0]	0.060
KOOS			
Pain [median (IQR)]	41.7 [36.1, 52.1]	41.7 [30.6, 52.8]	0.780
Symptoms [median (IQR)]	35.7 [21.4, 49.1]	46.4 [38.4, 61.6]	0.007
Activities of daily living [median (IQR)]	38.2 [29.4, 46.7]	41.2 [32.0, 50.0]	0.399
Sports [median (IQR)]	2.5 [0.0, 10.0]	10.0 [5.0, 15.0]	0.056
Quality of Life [median (IQR)]	18.8 [6.3, 31.3]	25.0 [6.3, 31.3]	0.854
FFI			
FFI pain [median (IQR)]	0.0 [0.0, 16.7]	0.0 [0.0, 19.4]	0.959
FFI function [median (IQR)]	0.0 [0.0, 33.6]	0.0 [0.0, 34.7]	0.889
FFI sum [median (IQR)]	0.0 [0.0, 50.3]	0.0 [0.0, 66.1]	0.982
AOFAS [mean (sd)]	75.0 [67.0, 81.5]	76.0 [68.0, 77.0]	0.820

Negative ROM values correspond to a motion deficit. Wilcoxon signed rank test for non-parametric data, Exact Fisher test for categorial variables. The significance level was *p < *0.05

*mTFA*   mechanical tibiofemoral angle, *ROM*   range of motion. *KOOS*   knee osteoarthritis outcome score, *FFI*   foot function index, *AOFAS*   American orthopedic foot and ankle score

Radiologic analysis confirmed that the preoperative mTFA significantly and negatively correlated with the preoperative HAVA (Pearson’s correlation = -0.72, *p < *0.001, Fig. 4). The results of the ie and ap AA testing, comparing valgus and varus, are reported in Table 2. Additional subanalysis of only valgus patients demonstrated that there were no significant differences between any of the four groups (0–5°, 5–10°, 10–15°, > 15°) with regard to ie rotation or ap displacement. Subanalysis of only varus patients did not demonstrate significant differences between the four groups using pairwise testing.

**Table 2:** Measurements of the ankle arthrometer could not show any significant differences between patients with preoperative varus and valgus osteoarthritis

	Valgus	Varus	*p*
*N*	30	52	
Inversion rotation (°) [median (IQR)]	41.4 [31.2, 45.8]	35.9 [30.3, 41.6]	0.174
Eversion rotation (°) [median (IQR)]	19.0 [16.0, 25.6]	21.0 [17.7, 25.3]	0.382
Anterior displacement (mm) [median (IQR)]	13.44 [9.9, 16.6]	11.2 [9.6, 16.8]	0.481
Posterior displacement (mm) [median (IQR)]	7.20 [4.6, 8.5]	5.6 [4.6, 8.0]	0.326

Further subanalysis of either valgus or varus patients using pairwise Wilcoxon testing could not show differences between the different groups (0–5°, 5–10°, 10–15°, > 15°) concerning inversion–eversion rotation or anterior–posterior displacement. Wilcoxon signed rank test, significance level *p < *0.05

**Fig. 5:**
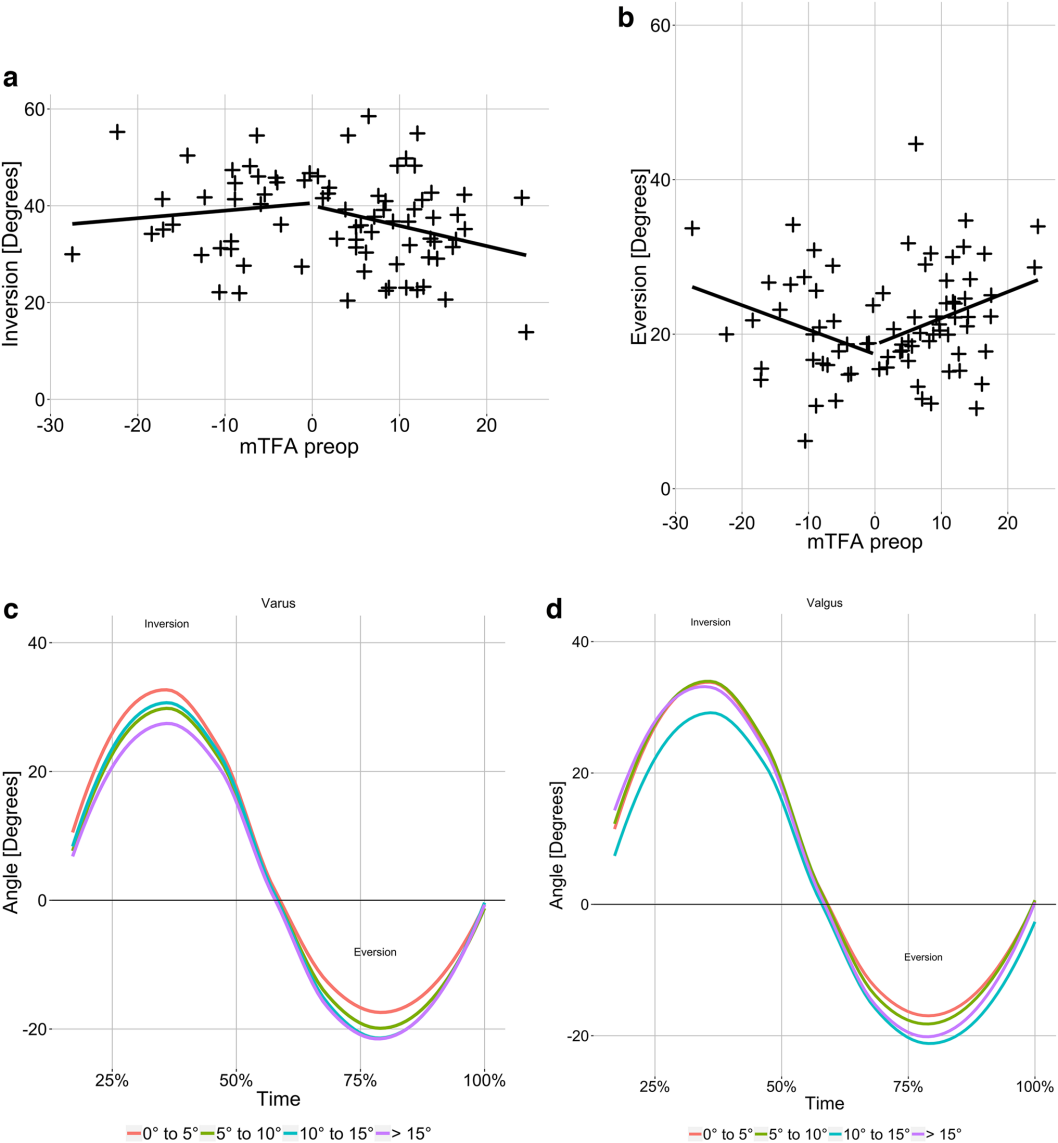
**a**–**b** The maximum inversion negatively correlated with the preoperative mTFA in both varus [cor =  − 0.24 (*p = *n.s.)] and valgus [cor = 0.11 (*p = *n.s.)] osteoarthritis. The maximum eversion positively correlated with the preoperative mTFA in varus [cor = 0.27 (*p = *n.s.)] and valgus [cor =  − 0.3 (*p = *n.s.)] osteoarthritis. Positive mTFA values = varus deformity, negative mTFA values = valgus deformity at the knee joint. Pearson’s correlation coefficient. **c**–**d** Line graphs visualising the inversion–eversion measurements on a percentage time scale. When the maximum inversion was reached, the AA was returned to baseline and then rotated into eversion. Pairwise Wilcox testing between all four groups revealed no significant differences between the maximum inversion and eversion. *mTFA* mechanical tibiofemoral angle. Significance level *p < *0.05

Testing of the laxity of medial and lateral ankle ligaments using the AA revealed that in both varus and valgus knee osteoarthritis, higher grades of preoperative deformity were not associated with changes in the ligamentous stability of the ankle joint (Fig. 5). Similarly, neither the MPTA nor the mLDFA significantly correlated with inversion or eversion in both the varus and valgus groups.

Correlation analysis demonstrated that the anterior displacement significantly correlated with the ADTA. Consequently, when the “slope” of the distal tibia (ADTA) became more horizontal, the posterior shift of the talus in relation to the tibia plafond increased (Fig. 2). Further analyses could not show any correlation between the mTFA and ADTA, between the knee slope and ADTA or between the anterior displacement and the knee slope or the posterior displacement and the knee slope. The preoperative mTFA did not correlate with the anterior or posterior displacement.

The intraobserver ICCs were 0.985 and 0.986 for the mTFA, 0.906 and 0.809 for the HAVA, 0.82 and 0.823 for the knee slope, 0.723 and 0.744 for the ADTA, 0.878 and 0.821 for the MPTA and 0.821 and 0.827 for the mLDFA. The interobserver ICC was 0.976 for the mTFA, 0.826 for the HAVA, 0.764 for the knee slope, 0.701 for the ADTA, 0.787 for the MPTA and 0.782 for the mLDFA.

## Discussion

The most important findings of this study were that in both varus and valgus knee osteoarthritis, increasing grades of frontal deformities were not associated with higher grades of ankle instabilities. Second, this report demonstrated for the first time that the bony morphology of the distal tibia plafond influences sagittal ankle joint stability.

Varus and valgus osteoarthritis of the knee are not only deformities of the frontal plane but are also known to influence the rotational alignment of the lower leg. In both types of deformities, increasing grades of mTFA were shown to be associated with higher grades of external rotation of the tibia [17, 25]. These multiplane changes can influence the soft tissue balance of the knee joint. In varus knee osteoarthritis, the attenuation of lateral soft tissue can cause lateral joint laxity, and in valgus knee osteoarthritis, the attenuation of medial soft tissue can cause medial joint laxity [6]. Whether an additional medial soft tissue contracture, e.g. in varus osteoarthritis, adds to lateral joint laxity is critically discussed [29, 35].

Contrary to the relationship between lateral and medial knee joint laxity in varus and valgus knee osteoarthritis, the laxity of the ankle joint was not affected by the mTFA in either varus or valgus patients. The hypothesis of this study was that in varus knee osteoarthritis, because of the compensation mechanism of the hindfoot shifting into eversion, the lateral soft tissue of the ankle joint became contracted, and medial soft tissue was attenuated. In valgus knee osteoarthritis, through hindfoot inversion, lateral soft tissue became loose, and medial ligaments contracted. However, the correlation between the mTFA and eversion/inversion of the ankle joint was weak and not significant for either group. Therefore, this part of the main hypothesis could not be confirmed. In contrast, the correlation between the mTFA and HAVA was strong and significant. One reason for that mismatch could be that the different forces on ligament tension at the ankle joint complex neutralise each other. In varus knee osteoarthritis, the ankle joint is oriented in varus [7]. Subsequently, the lateral collateral ligaments at the ankle joint could be under stress, and medial ligaments could be contracted. The eversion position of the hindfoot in varus knees might then work as a counteract mechanism and neutralise these forces with a higher stress on medial ligaments and reduction of lateral ligament tension. Similarly, the same mechanism working vice versa could explain why this study did not report ankle instabilities in knee valgus osteoarthritis. In summary, the compensation mechanism of the hindfoot, shifting into eversion in varus patients and into inversion in valgus patients, might have prevented ligamentous instabilities in the ankle joints analysed in this study.

Hubbard et al. examined 15 patients with knee osteoarthritis in their case–control study using an identical AA used in this study [15]. They found that patients with knee osteoarthritis had a significantly decreased ie rotation as well as decreased ap displacement compared to their matched controls. The present study did not include a control group as part of the study protocol. Therefore, differences between healthy controls and patients with knee osteoarthritis concerning ankle stability could not be derived. However, this study demonstrated that the ie rotation and ap displacement of the ankle joint were not affected by frontal deformation at the knee joint.

Recently, studies reported that TKA might lead to the onset or progression of ankle pain [7, 8, 10, 20]. However, these studies were conducted retrospectively. Consequently, the reported FFI values could be compared to the preoperative status. The present study fills that knowledge gap since it could be reported that the median FFI was zero in all three categories for both varus and valgus patients. One mechanism suggested for the phenomenon of increased ankle pain after TKA was that chronic hindfoot eversion to compensate for pathological varus knee alignments could result in a reduced ROM of the subtalar joint being fixed in an eversion position. A chronic hindfoot inversion to compensate for valgus knee deformities could result in a reduced ROM of the subtalar joint being fixed in an inversion position. The sudden correction of the mechanical leg axis after TKA could then lead to pathological pressure distributions in the ankle joint because the hindfoot could not return to a neutral position, ultimately causing ankle symptoms. In this study, the preoperative inversion and eversion capacity of the hindfoot was tested using an established AA. The results of the AA measurements demonstrated that a fixed inversion or inversion position of the hindfoot was not present in any of the cases (Fig. 5). In this study, the median FFI was zero for all three categories in both the varus and valgus groups. Recent publications discussed whether patients with increased ankle pain after TKA already had ankle symptoms before TKA, but these symptoms were masked by the pain caused at the knee joint [34]. In future investigations of this longitudinal study, the FFI will be assessed.

The sagittal alignment of the ankle joint in knee osteoarthritis could also have an impact on ankle symptoms and function. This study may prove, for the first time, that there is an association between the sagittal morphology of the distal tibia and an increased posterior displacement of the ankle. It remains to be clarified in future studies whether the ADTA changes after TKA and whether this has an impact on ankle symptoms. The intra- and interobserver reliability concerning the ADTA were merely moderate in this study but comparable to those values published in the literature [32]. Acquiring a clean lateral radiograph of the ankle joint, particularly in patients with high-grade frontal deformities, remains difficult, rendering a moderate reliability between observer measurements. This fact must be kept in mind when interpreting the abovementioned association between the ADTA and posterior displacement. The interrater reliability of the mTFA, tibial slope and HAVA were “excellent”, “good” and “good”, respectively, and had similar values as in other published studies [1, 12, 27, 30].

The limitation of this study was that in clinical ie testing of the hindfoot using the AA, movements are mostly a combination of the ROM of the subtalar joint and the medial/lateral soft tissue tension [9]. Therefore, the ie testing results in this or any other study do not show the laxity of ligaments or ROM of the subtalar joint separately, but rather as a combination of both. However, because the results from the ie testing showed values comparable to the literature of patients with a flexible subtalar joint, it can be assumed that in this study cohort, preoperatively, patients did not present with significantly reduced ROM of the subtalar joint [15]. Future studies could report ROM of the subtalar joint and ankle ligamentous stability separately by examining these movements with an image intensifier.

The results from this study are an important starting point for future research on the interaction between frontal knee deformities and the correction thereof. It has yet to be elucidated why TKA can induce ankle pain. Although the mTFA did not significantly correlate with inversion or eversion, higher grades of both varus and valgus deformities showed a tendency toward increased eversion and decreased inversion. The main reason this study could not detect ligamentous instabilities at the ankle joint can probably be attributed to a functioning subtalar joint, which neutralises forces on ligament tension at the ankle joint. Therefore, clinical examination before TKA should address the ankle joint complex to detect a reduced ROM of the subtalar joint, which can be treated by physiotherapy.

## Conclusion

This study could not confirm that high-grade frontal knee deformities were associated with ligamentous instabilities at the ankle joint or a fixed ROM of the subtalar joint. A horizontally aligned tibia plafond was associated with an increased posterior translation of the talus.

## Abbreviations

AA: Ankle arthrometer
ADTA: Anterior distal tibia angle
AOFAS: American orthopedic foot and ankle score
ap: Anterior–posterior
FFI: Foot function index
HAVA: Hindfoot alignment view angle
ie: Inversion–eversion
KOOS: Knee osteoarthritis outcome score
mTFA: Mechanical tibiofemoral angle
mLDFA: Mechanical lateral distal femur angle
PDTA: Posterior distal tibia angle
ROM: Range of motion
TKA: Total knee arthroplasty
